# The underestimated problem of oral *Candida* colonization—An observational pilot study in one nursing home

**DOI:** 10.1002/cre2.238

**Published:** 2019-09-10

**Authors:** Hannah Elisa Kottmann, Sonja Henny Maria Derman, Michael Johannes Noack, Anna Greta Barbe

**Affiliations:** ^1^ Centre of Dental Medicine, Department of Operative Dentistry and Periodontology University of Cologne Köln Germany

**Keywords:** nursing homes, oral hygiene, yeasts

## Abstract

**Objectives:**

Older people are at increased risk of intraoral yeast colonization. In this observational case series, we assessed *Candida* colonization among nine nursing home residents to investigate possible correlations with their individual characteristics, general health parameters, and oral care. We also described the effect of professional dental cleaning (PDC) including prosthesis cleaning on colonization cases.

**Materials and methods:**

General clinical and oral health was assessed in nine residents, and samples were taken from six oral mucosa sites or prosthetic surfaces. PDC was performed to achieve macroscopically clean results, and residents were re‐examined 2 weeks later.

**Results:**

We found that six residents were intraorally colonized with *Candida albicans*; four also had *Candida glabrata*. Prostheses were particularly infected. Dementia, multimorbidity, and presence of prostheses reduced oral hygiene ability; requiring assistance for oral hygiene care was a risk indicator for *Candida* colonization. PDC reduced *C. albicans* (at the expense of increased *C. glabrata*) but was not optimal for maintaining *Candida* reduction.

**Conclusion:**

In this pilot study, *Candida* colonization is prevalent among nursing home residents, especially those with cognitive impairment, multimorbidity, or reduced oral hygiene capacity. Potential negative effects on general health necessitate diagnostic and therapeutic guidelines. PDC alone did not maintain the reduction in *Candida* colonization; additional methods for daily oral care are necessary.

## 1 INTRODUCTION

Factors such as decreasing immune function, low salivary flow rates, or wearing prostheses can increase the risk of intraoral yeast colonization in older people.(Adachi, Ishihara, Abe, & Okuda, 2007; Adams, 1996) Poor oral hygiene is another risk factor for *Candida* colonization, (Arendorf & Walker, 1987) particularly in nursing homes, where the oral hygiene of residents is often insufficient. (Barbe, Kottmann, Derman, & Noack, 2019) This is partly due to lack of knowledge of good oral hygiene practices and high workload among nursing staff(Barbe, Kottmann, Hamacher, Derman, & Noack, 2019) and the cognitive limitations of residents.

*Candida albicans* and many other *Candida* species are part of the physiological human intraoral microbial flora(Bardia et al., 2019); their detection is not necessarily associated with disease. However, various pathological conditions have been associated with individual aspects of candidiasis. It is currently believed that the most common species causing systemic candidiasis is *C. albicans*,(Beighton, Hellyer, Lynch, & Heath, 1991) closely followed by *Candida glabrata*.(Brodin & Davis, 2017; Budtz‐Jlrgensen, Mojon, Banon‐Clement, & Baehni, 1996) Oral candidiasis occurs on the bottom of an uncontrolled proliferation of yeasts in the oral cavity, so local infections can lead from oropharyngeal candidiasis to systemic candidiasis.(Budtz‐Jorgensen, Mojon, Rentsch, & Deslauriers, 2000) An association exists between colonization of the oral cavity with *C. glabrata* or *Candida dubliniensis* and a low body mass index, interpreted as a sign of a limited general health status.(El‐Solh, 2011) Recent studies also show that oropharyngeal colonization with *Candida* and other yeasts poses a threat to the respiratory system, increasing the occurrence of pneumonia in general and aspiration pneumonia in particular.(Gupta, Gupta, & Varma, 2015; Haron, Vartivarian, Anaissie, Dekmezian, & Bodey, 1993; Hoad‐Reddick, Grant, & Griffiths, 1990; Holbrook & Hjorleifsdottir, 1986; Konsberg & Axell, 1994) Aspiration pneumonia is the most common cause of death among older people, and oropharyngeal aspiration is the most important factor that can trigger such pneumonia. This is mainly due to the progression of neurological diseases, noticeable in patients by the lack of coughing irritation and difficulty swallowing.(Kontoyiannis et al., 2002)

In recent years, high germ counts and documented oral candidiasis have been reported in 9–65% of nursing home residents,(Arendorf & Walker, 1987; Marik & Kaplan, 2003; Masur, Rosen, & Armstrong, 1977; Narhi, Ainamo, & Meurman, 1993; Patil, Rao, Majumdar, & Anil, 2015; Pfaller et al., 2004; Pound, Drew, & Perfect, 2002; Quagliarello et al., 2005) and it is well known that *Candida* infection in nursing homes exists. However, it seems that the problem and treatment strategies—despite the obvious impact—are not in the focus of dentists in daily clinical practice. A preventive oral hygiene program, consisting of regular tooth cleaning and oral hygiene instructions (recall intervals up to a maximum of 6 months), reduced the occurrence and germ counts of *Candida*.(de Resende, de Sousa, de Oliveira, Koga‐Ito, & Lyon, 2006) It remains unclear what direct effect oral hygiene measures such as professional dental cleaning (PDC) have on germ counts, occurrence, and selection of species of *Candida* yeasts in the oral cavity of residents of nursing homes.

In our recent study, the results of PDC by external dental professionals among nursing home residents were evaluated.(Rodriguez, Rex, & Anaissie, 1997; Ruhnke, 2006) In a subgroup of this study population, we also documented possible *Candida* colonization to indicate the overall infestation of *Candida* in nursing home residents and to record the impact of PDC on *Candida* manifestation and thus justify further studies with larger numbers of volunteers. Accordingly, species and colony numbers before and 2 weeks after PDC were characterized by microbiological examination, clinical characteristics, oral health, and oral hygiene habits of the residents. On the basis of clinical experience, among nursing home residents with dementia and multimorbidities, we hypothesize that we would not find any residents without *Candida* colonization. This case series, presented here, should also indicate possible patient‐related risk factors for *Candida* colonization. In the long term, this might lead to increased sensitivity for this problem and better identification of nursing home residents with an increased risk for systemic candidiasis.

## 2 MATERIALS AND METHODS

### 2.1 Subjects

Our case series included nine volunteers who lived in a retirement home (Bornheim, Germany) between December 2016 and March 2017. These were the last nine subjects that had been included in a randomized clinical trial investigating PDC performed by an external dental professional in a retirement home.(Ruhnke, 2006) Those additional oral health parameters reported in this study were not part of the initial study design. They were additionally assessed due to the obvious necessity to document the resident's candidiasis and prostheses colonization situation encountered in the overall study clinical trial. They were not supposed to give external validity with regard to the entire population of nursing home residents but were supposed to describe problems in individuals as precisely as possible because the group of nursing home residents is very heterogeneous. All subjects who agreed to the study were checked for exclusion criteria (less than four remaining teeth, foreseeable loss of remaining teeth due to diagnosed inflammation, or life‐threatening diseases at risk of dying soon). Before participating in the study, all subjects or their legal guardians provided written informed consent.

### 2.2 Study design

The general clinical and oral health status (tooth status, prosthetic situation, and periodontal status) was assessed for each subject. Personal parameters (number of months the subject had lived in the facility, oral care practices, that is, self‐cleaning or cleaned by the nurse, and nursing degree) and general health parameters (prescribed medication, systemic diseases, and cognitive status) were documented using medical information in the subjects' files at the nursing home. Decisive for the classification into a nursing degree according to German law is the degree of independence. In the process, the whole person and mental restrictions should be considered. There are five levels of care, and long‐term care is calculated by the number of points determined from a detailed set of questions for which an expert asks 64 criteria.

Subjects were examined at baseline (BL), and samples were taken with ESwabs™ (Copan Diagnostics) from six oral mucosa sites or prosthetic surfaces, if present. Subsequently, PDC was performed until macroscopically clean results were achieved. Two weeks later, the subjects were re‐examined (follow‐up [FU]) and samples taken using ESwabs, as before.

### 2.3 Procedure in the retirement home

All subjects received PDC to reach standardized prerequisites for oral hygiene. At study start, the dental nurse (HK) prepared all necessary materials (scalers, toothbrushes, ultrasound equipment for tooth cleaning, and interdental brushes) in the central bathroom of the nursing home. The subjects were visited by dental staff in their rooms and accompanied to the bathroom. Once the subjects were seated, the examination began. PDC was performed with all necessary aids permitted by the subject until a macroscopically clean situation was achieved. Prostheses were cleaned with a new manual brush and clean water.

### 2.4 ESwabs

The first and second ESwab was obtained from the inner surface of the upper and lower prosthesis. If no prosthesis was present, the swab was taken from the upper and lower anterior third of the palate or floor of the mouth. The third and fourth swabs were taken from the right and left vestibule, the fifth and sixth swabs from the right and left cheek mucosa. The ESwabs were stored at 4–8°C for a maximum of 24 hr.

### 2.5 Laboratory procedure

To allow the examiner to determine *C. albicans* and non‐*C. albicans* species, 0.5 ml of each ESwab was spread on a suitable CHROMagar™ plate. On the basis of the coloration, *C. albicans*, *C. dubliniensis*, *Candida krusei*, *C. glabrata*, and *Candida tropicalis* could be clearly determined. Different reactions of species‐specific enzymes with chromium as substrate allowed the growth and expression of specific colors. After 48 hr of incubation at 37°C, the colonies were determined by color and appearance. The absolute number of colonies was counted. The extent of culture growth was determined using the following scale: 0 colony‐forming unit (CFU) = no *Candida* growth; 1–20 CFU = subject carries *Candida*; 21–100 CFU = subject is infected with *Candida*; and >100 CFU = subject is highly infected with *Candida*.(de Resende et al., 2006)

### 2.6 Statistical analysis

The data were analyzed descriptively: Absolute and relative frequencies were assigned for qualitative variables, and the mean (standard deviation [SD]) was used for quantitative variables. All calculations were performed with SPSS Statistics 24 (IBM Corp. Armonk, NY, USA). The data were entered twice and revised for inconsistency.

## 3 RESULTS

### 3.1 Clinical characteristics

Nine nursing home residents or their legal guardians gave their written informed consent. The mean age was 85 years (SD: 10 years), and the mean number of months the respondent had lived in the home was 6 months (SD: 6 months). The mean number of preexisting conditions was 7 (SD: 2), and active components in the prescribed drugs was 12 (SD: 3). During the oral clinical examination, the subjects had an average of 9 (SD: 9) remaining teeth. The mean Decayed/Missed/Filled‐Teeth Index was 16.44 (SD: 7.18), and no change was observed during the study. One subject (11.1%) had a total prosthesis in the mandible, three subjects (33.3%) had partial dentures in the mandible, and two subjects (22.2%) had partial dentures in the maxilla. Four subjects (44.4%) did not wear a prosthesis. Eight subjects (88.9%) suffered from periodontitis.

### 3.2 Case descriptions according oral hygiene habits

#### 3.2.1 Oral hygiene performed by the nursing staff (*N* = 3)

Case 1 This subject was demented and multimorbid, had been living in the nursing home for 7 months, required nursing degree V, and her general condition was deteriorating. She wore a total prosthesis in the upper jaw and a partial prosthesis in the lower jaw, which could easily be removed by the patient on request but was not done daily except on explicit request (Table 1).

**Table 1:** Clinical characteristics

Case	1	2	3	4	5	6	7	8	9
Diagnosis of dementia (yes[y]/no[n])	y	y	y	n	y	y	y	n	n
Gender (male [m] or female [f])	f	f	m	m	f	f	f	f	f
Age (years)	89	65	91	71	86	88	85	91	98
Mobile (m) or using wheelchair (w)	w	w	m	m	m	w	m	m	m
Nursing grade ([2]; [25]; [14]; [20]; [28])	5	5	3	2	4	5	2	2	3
Months living in the nursing home	7	2	3	11	3	6	10	5	21
Number of morbidities (*n*)	11	5	3	5	9	8	8	10	8
Number of prescribed medications (*n*)	6	8	5	11	4	12	5	1	12
Number of teeth (*n*)	7	20	3	23	6	20	30	16	6
Total prostheses in at least one jaw (yes[y]/no[n])	y	n	n	n	n	n	n	n	n
Partial protheses (yes[y]/no[n])	y	n	y	y	y	n	n	y	n
Prostheses can be removed independently (yes[y]/no[n])	y	—	y	n	n	—	—	y	—

ESwab results. At BL, *C. albicans* was colonized with 0 to >100 colonies that mainly affected the prosthesis surfaces in the upper and lower jaw. At FU, there was a large decrease in the number of colonies. The subject developed fatal pneumonia. There was no confirmed diagnosis of the responsible pathogen spectrum (Tables 2 and 3).

Case 2 This subject was also demented and multimorbid, had been living in the nursing home for 2 months, with nursing degree V, but had a good general condition. Due to her dementia, she either seldom swallowed or did not swallow at all, which meant that food and sweet drinks often remained in her mouth for hours. Her toothbrushing skills were zero, but she recognized what the toothbrush was. She could not rinse her mouth or spit it out.

**Table 2:** ESwabs and oral indices: GI, OHI, and VMI Proband at BL and FU 2 weeks after professional dental cleaning

Case	1		2		3		4		5		6	
	BL	FU	BL	FU	BL	FU	BL	FU	BL	FU	BL	FU
A	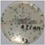	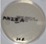	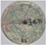	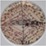	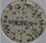	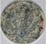	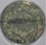	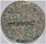	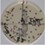	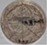	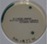	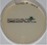
B	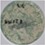	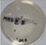	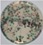	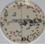	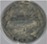	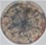	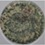	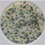	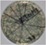	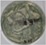	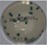	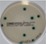
C	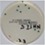	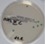	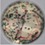	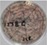	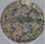	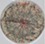	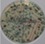	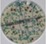	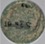	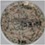	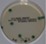	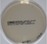
D	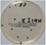	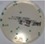	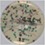	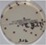	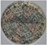	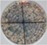	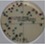	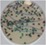	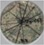	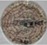	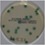	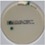
E	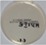	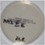	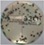	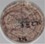	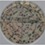	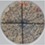	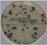	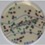	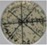	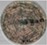	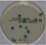	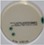
F	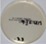	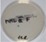	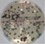	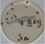	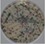	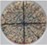	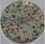	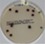	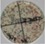	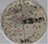	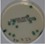	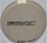
GI	3.0	1.0	3.0	2.0	3.0	3.0	3.0	3.0	—	—	3.0	3.0
OHI	4.0	3.0	8.0	1.5	6.0	3.0	8.4	5.0	4.0	4.0	11.7	4.7
VMI	9.5	0.0	6.0	2.8	8.0	0.0	11.0	0.0	15.0	0.0	10.0	0.0

*Note.* A, upper jaw prostheses; a, anterior third palate; B, lower jaw prostheses; b, anterior base of the mouth; C, vestibulum right; D, vestibulum left; E, right cheek mucosa; F, left cheek mucosa.

Abbreviations: BL, baseline; FU, follow‐up; GI, Gingivitis Index; OHI, Oral Hygiene Index; VMI, Volpe‐Manhold‐Index.

**Table 3:** Number of colonies of *Candida albicans* and *Candida glabrata* (A–F) at BL (before PDC) and FU (2 weeks after PDC)

	A		B		C		D		E		F	
*Candida*	*C. albicans*	*C. glabrata*	*C. albicans*	*C. glabrata*	*C. albicans*	*C. glabrata*	*C. albicans*	*C. glabrata*	*C. albicans*	*C. glabrata*	*C. albicans*	*C. glabrata*
Case 1												
BL	130	0	>100	0	10	0	9	0	0	0	0	0
FU	0	0	3	0	6	0	27	0	0	0	1	0
Case 1												
BL	>100	>100	227	154	356	206	130	42	66	48	66	48
FU	35	491	38	116	163	>800	1	30	238	>100	3	14
Case 3												
BL	39	155	L	L	108	770	261	>100	39	316	63	342
FU	535	680	575	>1000	1084	3500	567	3816	198	2600	170	1268
Case 4												
BL	L	L	<100	<100	369	30	36	15	42	15	190	50
FU	812	1136	199	69	253	20	106	71	52	47	4	12
Case 5												
BL	20	97	>100	>100	>100	>100	840	<100	163	300	86	>100
FU	43	>100	>100	>100	201	800	288	980	800	>100	43	165
Case 6												
BL	2	0	24	0	8	0	22	0	12	0	17	0
FU	1	0	8	0	0	0	3	0	3	0	0	0

*Note.* A, upper jaw prostheses; a, anterior third palate; B, lower jaw prostheses; b, anterior base of the mouth; C, vestibulum right; D, vestibulum left; E, right cheek mucosa; F, left cheek mucosa; L, lane. Colors: none (green) = 0 CFU; carrier (yellow) = 0 to 20 CFU; infected (orange) = 21–100 CFU; massively infected (red) = over 100 CFU.

Abbreviations: BL, baseline; CFU, colony‐forming units; FU, follow‐up; PDC, professional dental cleaning.

ESwab results. At BL, *C. albicans* and *C. glabrata* were found, with lower *C. glabrata* colony numbers. At FU, *C. albicans* values on the dentures decreased, but *C. glabrata* colony counts either only slightly decreased or increased; four out of six sites were colonized with significantly fewer *Candida* colonies than at BL. The high *Candida* values in the oral cavity could be an indication that oral hygiene measures were almost completely abandoned in this patient. Her dementia made it almost impossible for nursing staff to brush the teeth in the time specified, which could explain the high infestation. Furthermore, it is possible that PDC had an effect on the habitat of *C. albicans*, at the expense of a niche becoming available for increased colonization by *C. glabrata*.

Case 3 This subject was a male dementia sufferer and had been living in the nursing home for 3 months, required nursing degree III, and had a good general condition. He had partial prostheses in his upper and lower jaw, which he could remove himself. Also, he showed intraoral mucous membrane bleeding and swelling with pain compatible with the diagnosis of intraoral candidiasis.

ESwab results. At BL, large quantities of *C. albicans* and *C. glabrata* were found, with the highest values on the prostheses. At FU, *C. albicans* had increased at almost all measuring points, whereas the number of colonies of *C. glabrata* increased massively (similar to Case 2). Despite his advanced dementia, the subject appeared very self‐reliant and self‐confident. It is uncertain whether he was assisted by nursing staff when brushing his teeth or whether there was only a monitoring/reminder. Whether the prosthesis was taken out of the mouth during the day or at night could not be confirmed. It is conspicuous that the subject sometimes exceeded 10 to >30 times the amount of *Candida* colonies needed to be considered massively infected. Again, it may be that PDC allowed new niches for *C. glabrata* to be created by the possible displacement of *C. albicans*. This subject also developed fatal pneumonia. A reliable diagnosis of the responsible pathogen spectrum is not available.

#### 3.2.2 Self‐cleaning without external support

Case 4 This subject was a male suffering from Parkinson's disease, had been living in a nursing home for 11 months, required nursing degree II, and had a good general condition. He wore an upper jaw partial prosthesis that could not be removed by himself or nursing staff. His ability to brush his teeth was reduced, but he had not yet received help with oral hygiene. Also, he showed intraoral mucosal swelling with pain localized under the upper jaw prosthesis compatible with the diagnosis of intraoral candidiasis.

ESwab results. At BL, *C. albicans* and *C. glabrata* were present, particularly on the dentures. High values on the maxillary prosthesis could be explained by the fact that the subject had not removed it for 2 weeks. *C. albicans* was initially represented with higher colony numbers. At FU, higher numbers of *C. glabrata* than *C. albicans* were found on the prosthesis. In general, no significant reduction of candidiasis was achieved by PDC in this subject. The subject developed fatal pneumonia. A reliable diagnosis of the responsible pathogen spectrum was not available.

Case 5 This subject was demented and multimorbid. She had been living in the nursing home for 3 months, required nursing degree IV, and was in a bad general condition. She wore an upper and lower partial prosthesis, which she and the nursing home staff could not remove. She had insufficient tooth brushing skills and never brushed her teeth on her own, unless asked to do so.

ESwab results. At BL, five out of six oral sites were massively infected with *Candida*. Both *C. albicans* and often higher counts of *C. glabrata* were present. At FU, there was no reduction in the high number of colonies after PDC. A change from a higher number of *C. albicans* to *C. glabrata* colonies was once again observed.

Case 6 This subject was demented and multimorbid, had been living in a nursing home for 6 months, required nursing degree V, and had a good general condition. She had no prostheses. Her toothbrushing ability was good, but as her dementia mainly affected her short‐term memory, she brushes her teeth on her own.

ESwab results. At BL, low CFU values of *C. albicans* were found, without colonization by *C. glabrata*. At FU, a significant reduction in the number of *Candida* colonies was observed, and it was assumed that PDC reduced *Candida* colonization.

Cases 7, 8, and 9 were female and lived in a nursing home for between 5 and 21 months. All were in good general condition and self‐reliant, with no cognitive limitations. Two were diagnosed with depression, and one was an alcoholic (one bottle of wine per day). One subject had a lower jaw prosthesis, and two had no prostheses. Two subjects (Cases 7 and 8) had good toothbrushing skills, but Case 9 did not; all three brushed their teeth on their own.
  ESwab results. No *Candida* species were found at BL or FU. The cognitive abilities and very good oral hygiene care (in two subjects) might have prevented colonization with *Candida*. Despite poor oral hygiene in Case 9, the presence of just five teeth in the mandibular front (an exposed position directly in front of the salivary gland) and the absence of prostheses may explain the absence of *Candida* species.

## 4 DISCUSSION

As in other studies, our observational case series provides indications that intraoral colonization with *C. albicans* and *C. glabrata* is highly prevalent among nursing home residents. Risk factors for *Candida* colonization in our observational data included dementia, multimorbidity, the presence of prostheses, and reduced oral hygiene ability, and an increased need for assistance in oral hygiene care appeared to be a risk indicator. Six of our subjects were intraorally colonized with *C. albicans*, and four of these also had *C. glabrata*; *C. glabrata* only occurred in combination with *C. albicans*. If *C. albicans* and *C. glabrata* were found together, usually, more colonies of *C. albicans* were present. Overall, a strong prosthesis infestation was observed; in two subjects with maxillary prostheses, lawn growth was documented. In these subjects, the intraoral examination also showed clinical signs of candidiasis (mucosal swelling, pain, and mucosal bleeding).

The effects of the mainly mechanical PDC that is performed in outpatient care is unclear. We did not observe a sustained effect of PDC on *Candida* colonization of oral mucosa and prostheses. Two weeks after PDC, there was no sustained reduction in germ counts in three out of four of subjects, whereas in two cases, the number of colonies was even higher than at BL. It appears that PDC had some positive effects on *C. albicans* colonization, but mechanical cleaning seemed to shift the ecological niches and lead to new and increased colonization by other *Candida* species such as *C. glabrata*. It is unclear exactly what PDC can achieve regarding *Candida* infestation and its effects, particularly regarding its risks versus its benefits.

Studies show that *C. albicans* is an effective respiratory pathogen associated with aspiration pneumonia.(Konsberg & Axell, 1994) Pneumonia is the most common cause of infection‐related deaths in people aged ≥65 and accounts for 13–48% of all infections in retirement homes.(Salerno et al., 2011) In our study, three subjects developed pneumonia, which led to their death. Poor oral hygiene appears to be the greatest risk factor for pneumonia in nursing home residents(Samaranayake, Lamb, Lamey, & MacFarlane, 1989); our subjects who died of pneumonia had reduced oral hygiene ability and were colonized with high CFU numbers of *C. albicans*. *C. albicans* has been described as the triggering pathogen for *Candida*‐induced pneumonia, but mixed infections with various pathogens such as *C. glabrata* have also been reported.(Beighton et al., 1991) Our case series also indicates that *C. albicans* is more likely to occur alone in cases of low infestation and in combination with *C. glabrata* in cases of very severe infestation. A systematic review has shown that one in 10 pneumonia‐related deaths in older people could be prevented by better oral hygiene and thus better oral health.(Schou, Wight, & Cumming, 1987) Our case series indicates that PDC does not play a central role, at least not with regard to *Candida* colonization; we hypothesize that PDC was more likely to create new ecological niches that allowed an increase in *C. glabrata* colonization. Thus, the benefits of PDC versus potential risks in this vulnerable group need to be determined. Recommendations for action and guidelines for dentists working in the nursing home are urgently required. Such recommendations already exist, for example, in cardiac surgery,(Sjogren, Wardh, Zimmerman, Almstahl, & Wikstrom, 2016) where the risk of postoperative pneumonia is reduced by approximately one half among patients receiving preoperative chlorhexidine mouthwash; they suggest adoption of this recommendation in preoperative protocols could help improve patient outcomes.

*Candida*‐associated prosthetic stomatitis has been found in 60–65% of prosthesis wearers in long‐term care as we could show among participants (Cases 3 and 4) with an upper and lower jaw prostheses with intraoral clinical signs of candidiasis. It is described as the most common form of oral candidiasis.(Arendorf & Walker, 1987) Prosthetic stomatitis causes mucous membrane bleeding, dry mouth, swelling, taste disorders, and burning mouth or pain sensations(Vigild, 1987; Wilkieson, Samaranayake, MacFarlane, Lamey, & MacKenzie, 1991). In the vulnerable group of older people with dementia, who may not be able to express pain, this is a problem that can lead to reduced food intake. The accumulation of microbial biofilm on the prosthesis surface and its contact with the oral mucosa is a local predisposing factor that can lead to the development of prosthesis stomatitis.(Arendorf & Walker, 1987) The high colonization of *Candida* in the oral cavity can also lead to an oropharyngeal spread of *Candida* infection and promote systemic candidiasis.(Budtz‐Jorgensen et al., 2000) In four out of five of our subjects who had prosthesis, *Candida* was present in high colony numbers. In three of these four subjects, the partial (mainly lower jaw) prostheses had the highest *Candida* count compared with the entire oral cavity. Although lower jaw partial prostheses rarely cause prosthetic stomatitis,(Zakaria et al., 2017) they make a large contribution to the total amount of *Candida* in the oral cavity. Our results provide first indications that partial prostheses represent a hygiene problem and should therefore become a focus of clinical investigations.

Further studies are needed to clarify possible risk factors that predispose subjects to systemic *Candida* infections in oral *Candida* occurrences. In our cases, there were indications that residents with dementia, multimorbidity, limited oral hygiene ability, and dental prostheses had the highest *Candida* levels; this should be confirmed in studies with larger case numbers. It is known that multimorbidity and polymedication worsen oral health, as do the presence of dementia and lack of mobility.(Zenthofer et al., 2017; Zenthofer, Schroder, Cabrera, Rammelsberg, & Hassel, 2014)

In the long term, it is possible that nursing home residents at risk could benefit from *Candida* screening of the oral cavity, according to MRSA screening at every hospital admission. When they move into the nursing home, they undergo an intensive medical check‐up; currently, microflora screening is not part of this routine. If the need for assistance is not obvious due to previous illnesses, the extent of the lack of oral hygiene skills is often not recognized. Such investigations could not only detect *Candida* colonization but also potentially help prevent pneumonia and systemic candidiasis. Considering how easily a swab can be taken from the mucosa within a few seconds, this procedure is suitable for routine examinations. We confirm that the use of ESwabs for *Candida* screening is simple and effective in assessing the risk of oral candidiasis and could therefore help to prevent clinical complications such as pneumonia in elderly residents by providing evidence of necessary assistance, dental treatment, or drug intervention.

Our small number of participants (nine subjects) is responsible for the fact that our results are very limited and individual; as far as their significance and external validity is concerned, they cannot be applied to the entire population of nursing home residents. The group of nursing home residents is very heterogeneous and difficult to generalize. It should be discussed whether clinical research within this group requires more individual study designs with more precise patient descriptions, as few factors can be generalized within these groups.

The prevalence of *Candida* colonization is high among nursing home residents, especially those with additional risk factors (cognitive impairment, multimorbidity, and reduced oral hygiene capacity). The potential negative effects on general health suggest a need for diagnostic and therapeutic guidelines. PDC alone in this small study population did not maintain the reduction in *Candida* colonization; additional methods for daily oral care are necessary.

In summary, the small number of our study participants seems to correspond to the literature regarding the well‐known problem of oral *Candida* infection, which remains underestimated in daily dental care of nursing home residents, especially those with additional risk factors (cognitive impairment, multimorbidity, and reduced oral hygiene capacity) with possible negative effects on their general health and well‐being. Observational data of this case series suggest that PDC alone in this population cannot maintain the reduction in *Candida* colonization; additional methods for daily oral care are necessary, and the results underline the need for necessary diagnostic and therapeutic guidelines.

## CONFLICT OF INTEREST

None of the authors report any conflict of interest.

## ETHICS

The University of Cologne local ethics review board (16‐204) approved the study, which was registered in the German Clinical Trials register (https://www.germanctr.de; number DRKS00010767) before the first subject was included.
